# Regulating Pores and Carbonyl Groups of Biomass‐Derived Hard Carbon for Enhanced Sodium Storage

**DOI:** 10.1002/advs.202510328

**Published:** 2025-07-30

**Authors:** Chi Chen, Yapeng Tian, Run Ren, Shuocong Duan, Dandan Wang, Zhuosen Wang, Yunfeng Chao, Jianhua Zhu, Xinwei Cui

**Affiliations:** ^1^ Henan Institute of Advanced Technology Zhengzhou University Zhengzhou 450003 P. R. China; ^2^ Zhongyuan Critical Metals Laboratory Zhengzhou University Zhengzhou 450001 P. R. China

**Keywords:** carbonyl group, electrochemical kinetics, nanoconfinement, nucleation energy barriers, sodium‐ion batteries

## Abstract

This study investigates the impact of sulfuric acid‐assisted hydrothermal pretreatment on the structural and electrochemical properties of biomass‐derived hard carbon for sodium‐ion batteries. Advanced characterization demonstrates that sulfuric acid‐mediated hydrothermal strategy promotes the formation of crosslinked small molecules within the precursor. This process yields optimized hard carbon exhibiting pore‐orifice nanoconfinement, enriched C═O functional groups at pore interface, and increased proportion of closed pores after carbonization. Collectively, these structural refinements synergistic enable exceptional sodium storage performance through accelerated desolvation kinetics, reduced nucleation energy barriers, and enhanced closed pore utilization efficiency to boost specific capacity. Specifically, the SAHTC‐1300 anode delivers a remarkable reversible capacity of 386 mAh g^−1^ at 50 mA g^−1^, maintains 270 mAh g^−1^ at 2 A g^−1^, and retains 90% capacity after 1000 cycles at 1 A g^−1^, outperforming control samples (HTC‐1300 and SCG‐1300). The work demonstrates the efficacy of sulfuric acid‐assisted hydrothermal carbonization for synthesizing high‐performance biomass‐derived hard carbon anodes, offering valuable insights for the design of advanced biomass‐based energy storage materials.

## Graphical abstract

Sulfuric acid‐mediated hydrothermal carbonization for biomass‐derived hard carbon exhibits tailored pore orifices, along with the formation of abundant small‐sized closed pores and carbonyl groups localized at pore peripheries, thereby increasing the efficiency for sodium ions storage.

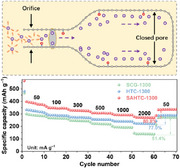

## 1 Introduction

The intermittent nature of renewable energy necessitates sustainable, cost‐effective energy storage systems, where lithium‐ion batteries (LIBs) dominate with high energy density and cycle life but face challenges of lithium scarcity and uneven distribution, prompting sodium‐ion batteries (SIBs) to emerge as a viable alternative leveraging sodium's abundance, lower cost, and comparable performance for sustainable energy storage solutions.^[^ ^1^ ^]^ A critical challenge for SIBs lies in identifying suitable anode materials. Graphite, the commercial anode for LIBs, exhibits poor sodium storage capability due to the larger ionic radius of Na^+^.^[^ ^2^ ^]^ In contrast, hard carbon (HC) has gained attention as a viable anode material owing to its tunable interlayer spacing, defect‐rich structure, and abundant micropores, which facilitate Na^+^ insertion and adsorption.^[^ ^3^ ^]^

Compared to graphite, the structural complexity of hard carbon results in ambiguous sodium storage mechanisms across its diverse configurations and significant progress has been made by researchers in understanding the complex sodium‐ion storage behavior within hard carbon microstructures, particularly in elucidating the sodium storage mechanisms associated with the slope region and plateau region of charge/discharge profiles.^[^ ^4^ ^]^ These established mechanisms evolve sequentially from an “intercalation‐adsorption” mechanism to an “adsorption‐intercalation” pattern, ultimately transitioning into a hybrid “adsorption‐intercalation/filling” mechanism,^[^ ^5^ ^]^ whose developmental trajectory provides critical guidance for optimizing hard carbon performance. The storage process is generally initiated by Na^+^ adsorption at defect sites and open pore surfaces, corresponding to the high‐potential slope region (>0.1 V) that exhibits rapid reaction kinetics. While strategies such as heteroatom/defect introduction have been employed to enhance slope region capacity and rate capability, these modifications typically compromise plateau capacity or reduce initial Coulombic efficiency (ICE).^[^ ^6^ ^]^ Subsequent sodium storage involves either or both of the following processes: Na^+^ intercalation within carbon interlayers and quasi‐metallic Na cluster filling in closed pores, which collectively contribute 60–90% of total capacity depending on closed pore dimensions and geometry. This highlights the critical role of closed pores in achieving high energy density. Although strategies such as open‐to‐closed pore conversion,^[^ ^7^ ^]^ defect minimization,^[^ ^8^ ^]^ and pore entrance size reduction^[^ ^9^ ^]^ effectively enhance plateau capacity or ICE, they inevitably sacrifice slope region capacity and rate performance. Notably, ≈40% of pores remain unutilized even after sodiation,^[^ ^10^ ^]^ suggesting substantial potential for total capacity improvement. Consequently, the fundamental challenge in optimizing sodium storage performance resides in achieving complete activation of these electrochemically inert closed pores while maintaining uncompromised rate capability, a persistent limitation that remains unresolved by current modification strategies due to mechanistic deficiencies in established approaches.

Herein, we propose a simple and efficient method for the pretreatment of biomass precursors to prepare HC materials with superior electrochemical performance. Ex situ structural analysis revealed that sulfuric acid‐assisted hydrothermal strategy can decompose cellulose and hemicellulose into smaller molecules during oxidative degradation, while simultaneously oxidizing hydroxyl groups on some monomer side chains into carboxyl groups. These carboxyl groups subsequently react with adjacent hydroxyl groups to form a highly crosslinked structure. The crosslinked structure contributes to maintaining structural stability during high‐temperature calcination and inhibits graphitization of carbon layers. The resulting hard carbon exhibits tailored pore orifices, along with the formation of more small closed pores and C═O groups at pore edges. These three factors synergistically enhance the Na^+^ storage performance and significantly improve the fast‐charging capability of the hard carbon. Electrochemical performance tests demonstrated that the SAHTC‐1300 electrode delivers an exceptionally high reversible capacity (386 mAh g^−1^ at 50 mA g^−1^), along with outstanding cycling stability (capacity retention of 90% after 1000 cycles at 1 A g^−1^) and remarkable rate capability (270 mAh g^−1^ at 2 A g^−1^). Furthermore, the sodium storage behavior of SAHTC‐1300 was investigated using in situ Raman spectroscopy and ex situ XPS. This study provides a novel pretreatment strategy for hard carbon precursors, offering crucial guidance for the development of high‐performance biomass‐derived hard carbon materials.

## 2 Results and Discussion

**Figure 1:**
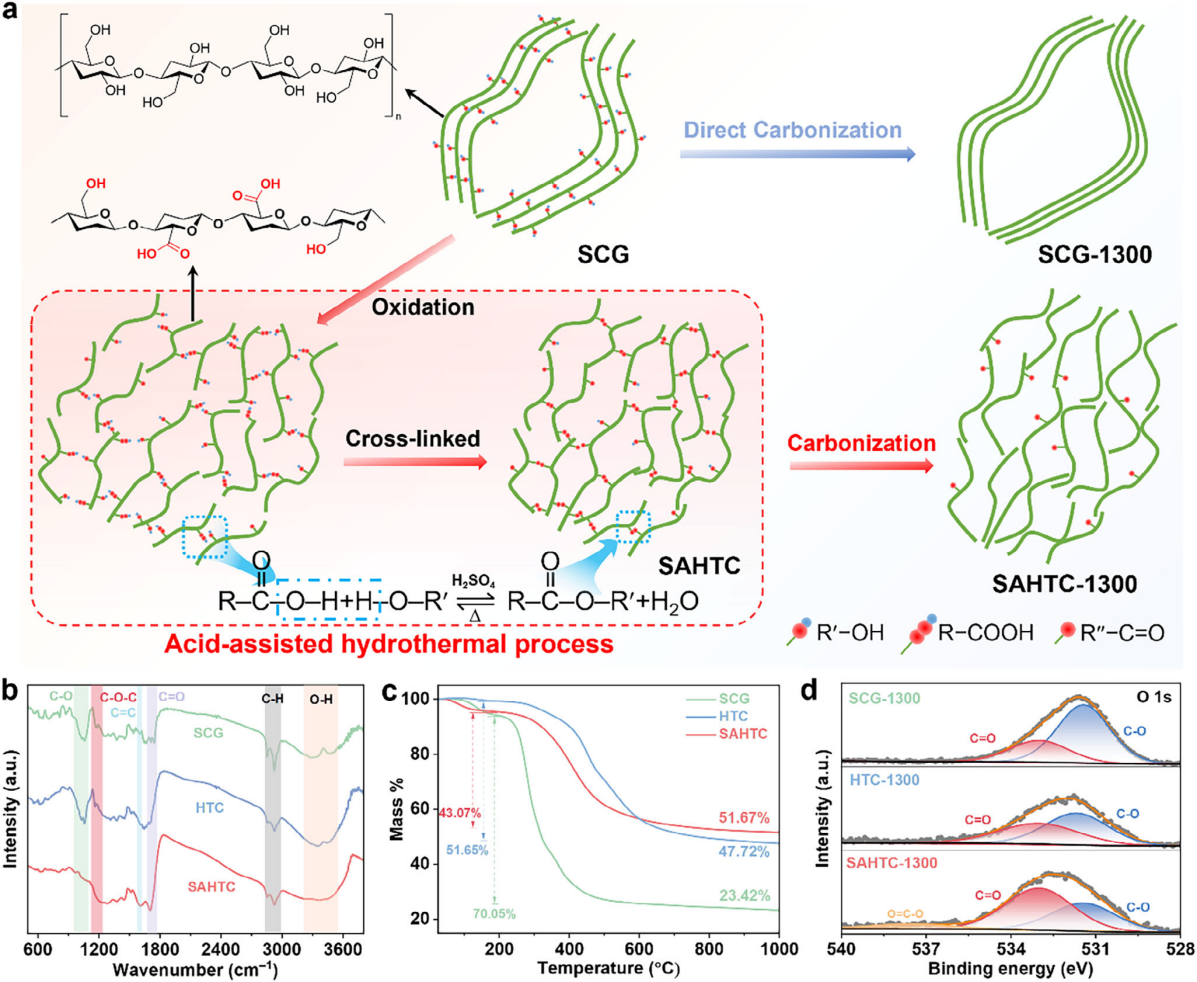
a) Schematic illustration of the synthesis process for SAHTC‐1300 and SCG‐1300. b) FTIR spectra and c) Thermogravimetric (TG) curves of SCG, HTC, and SAHTC. d) High‐resolution O1s XPS spectra of SAHTC‐1300, HTC‐1300, and SCG‐1300.

The synthetic pathway for sulfuric acid‐assisted hydrothermal carbon (SAHTC) is schematically illustrated in Figures S1 and S2 (Supporting Information). Initially, coffee grounds were homogeneously dispersed in a sulfuric acid solution and underwent hydrothermal pretreatment. The processed precursor was subsequently pyrolyzed at 1300 °C to obtain the SAHTC‐1300 material. To systematically assess the influence of sulfuric acid during hydrothermal pretreatment, two control samples were prepared that HTC‐1300 was synthesized via hydrothermal pretreatment (excluding sulfuric acid) followed by carbonization under identical conditions, and SCG‐1300 was derived from direct pyrolysis of raw coffee grounds without pretreatment (Figure S3, Supporting Information). As illustrated in **Figure** **1**a, the SAHTC strategy diverges from conventional direct pyrolysis through its dual‐stage pretreatment mechanism (oxidative decomposition and crosslinking). In the first stage, sulfuric acid catalyzes the hydrolysis of glycosidic C─O─C bonds in biopolymers, generating smaller molecular fragments while oxidizing hydroxyl groups on monomer side chains to carboxyl functionalities. These carboxyl groups subsequently undergo esterification with adjacent hydroxyl groups during the crosslinking stage, forming a robust 3D network. During high‐temperature carbonization, this crosslinked architecture evolves into a disordered graphitic framework with abundant closed pores. This method presents a streamlined and efficient alternative to conventional labor‐intensive pretreatment approaches, effectively eliminating impurities while simultaneously enhancing purification and boosting carbon yield, rendering it exceptionally suitable for large‐scale commercial applications.

To systematically evaluate the pretreatment‐induced evolution of functional groups, Fourier transform infrared spectroscopy (FTIR) analysis was conducted (Figure 1b; Figure S4, Supporting Information). A prominent broadband absorption feature at 3400 cm^−1^ corresponds to –OH stretching vibrations in methylene groups, while the well‐defined vibrational signature at 1059 cm^−1^ originates from pyran ring‐associated –OH stretching modes. Notably, the SAHTC exhibits a characteristic absorption band at 1705 cm^−1^, attributable to C═O stretching vibrations in carboxyl/ester moieties, unequivocally confirming carboxyl group formation. Concurrently, the C–O–C stretching mode at 1062 cm^−1^ demonstrated significant intensity enhancement, spectral broadening, and a red shift, while the weakening of –OH signals indicated esterification reactions, leading to cross‐linked structures.^[^ ^11^ ^]^ Additionally, the enhanced C═C absorption at 1600 cm^−1^ contributed to the overall structural stability. To analyze the structural changes during high‐temperature carbonization, thermogravimetric analysis (TGA) was performed (Figure 1c). The SAHTC‐derived hard carbon exhibited the highest carbon yield (51.67%), surpassing those of HTC (47.72%) and SCG (23.42%). Notably, SAHTC demonstrated the lowest weight loss (43.07%) in the temperature range of 250–1000 °C, significantly lower than those of HTC (51.65%) and SCG (70.05%). This demonstrates the exceptional structural stability of SAHTC, featuring substantially suppressed decomposition from side reactions during carbonization, stemming from its densely cross‐linked architecture. The high‐temperature carbonized hard carbons from three samples were characterized by X‐ray photoelectron spectroscopy (XPS) in Figure 1d and Figure S5 (Supporting Information), with all specimens displaying well‐defined C and O signatures. Deconvolution of O 1s spectra resolved three characteristic components of carbonyl oxygen (C═O at 533 eV), alkoxy oxygen (C‐O at 531.5 eV), and ester linkages (O‐C═O at 537.5 eV) formed through dehydration condensation.^[^ ^12^ ^]^ Crucially, SAHTC‐1300 exhibited markedly enhanced C═O content relative to counterparts, coupled with persistent O─C═O bonding. Furthermore, X‐ray photoelectron spectroscopy (XPS) analysis performed on the SAHTC‐1300 sample after 100 s of surface etching (Figure S6, Supporting Information) revealed a significant presence of C═O groups within the pore structure. This finding demonstrates that oxygen‐containing functional groups are not only distributed on the sample surface but are also preserved within closed pores during high‐temperature carbonization. These findings validate the progression of esterification processes, aligning with complementary FTIR observations (Figure S7, Supporting Information). Ex situ temperature‐dependent FTIR analysis (Figure S8, Supporting Information) documented progressive elimination of oxygen functionalities in SAHTC, whereas robust C═C and C‐O‐C vibrations‐maintained intensity during thermal treatment,^[^ ^13^ ^]^ confirming their superior thermal stability relative to other moieties, a critical factor in maintaining structural coherence that aligns with TGA‐derived stability parameters.

**Figure 2:**
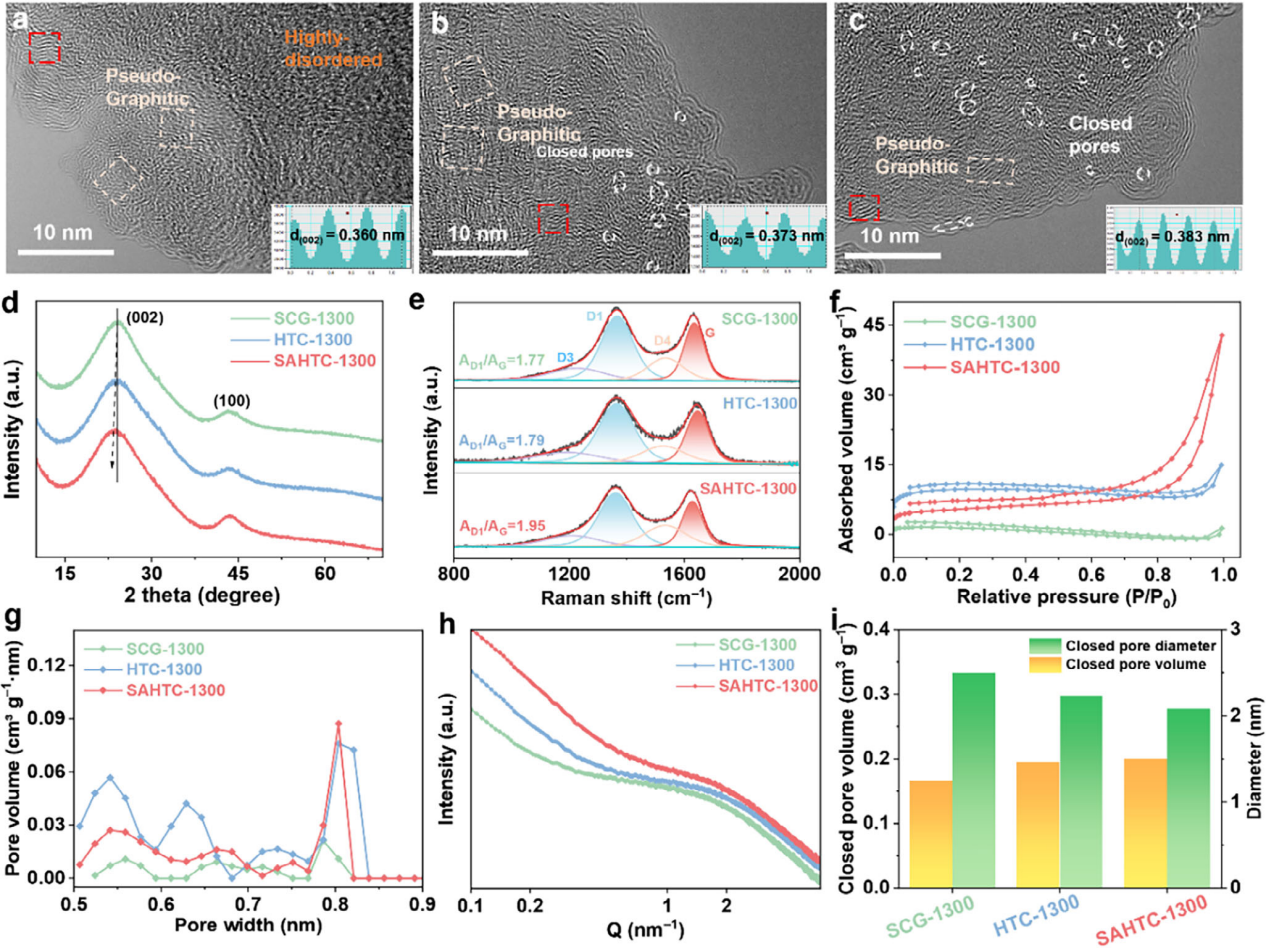
HRTEM images of a) SCG‐1300, b) HTC‐1300, and c) SAHTC‐1300. d) XRD patterns, e) Raman spectra, f) N_2_ adsorption‐desorption isotherms, g) CO_2_ pore size distribution, h) small‐angle X‐ray scattering (SAXS) patterns, and i) closed pore diameter and closed pore volume of SCG‐1300, HTC‐1300, and SAHTC‐1300.

High‐resolution transmission electron microscopy (HRTEM) images (**Figure** **2**a‐c) reveal the disordered structures within the different samples. Compared to SCG‐1300 and HTC‐1300, SAHTC‐1300 exhibits a larger interlayer spacing (d_002_ = 0.383nm) and higher porosity, which is in good agreement with the XRD and pore size distribution analyses. Additionally, the ring‐shaped pseudo‐graphitic structure accompanied by a significant number of nano‐voids is observed. These enclosed pores provide additional sites for sodium ion chelation, thereby contributing to the enhanced sodium ion storage capacity.^[^ ^14^ ^]^ The crystal structures of the samples are further analyzed by XRD (Figure 2d). The distinctive diffraction pattern characteristic of hard carbon materials emerges at ≈22° and 43°, corresponding to the (002) and (100) graphitic crystalline lattice planes. The (002) peak of SAHTC is positioned at a lower angle, indicative of a larger interlayer spacing. By fitting the XRD curves (Figure S9, Supporting Information), the proportions of the highly disordered region, the pseudo‐graphitic region, and the graphitic‐like region in hard carbon can be determined. The results indicate that HTC‐1300 exhibits the highest proportion of the highly disordered region among the three samples, which contributes to its increased capacity in the sloping region. In contrast, SAHTC‐1300 achieves the highest proportion of the pseudo‐graphitic region (70%) due to its cross‐linked structure, which facilitates well‐organized pseudo‐graphitic domain formation and enhances sodium ion storage.^[^ ^15^ ^]^ Raman spectroscopy (Figure 2e) was employed to assess the degree of graphitization of the samples. The peak observed at ≈1347 cm^−1^ corresponds to the D‐band, indicative of disordered defect structures within the graphite. Conversely, the peak near 1631 cm^−1^ represents the G‐band, signifying the presence of crystalline graphite structures. The main peaks in the Raman spectrum can be deconvoluted into four sub‐peaks, located at 1200 cm^−1^ (D4), 1369 cm^−1^ (D1), 1532 cm^−1^ (D3), and 1631 cm^−1^ (G).^[^ ^16^ ^]^ Generally, the degree of graphitization can be evaluated by the integral area ratio of A_D1_/A_G_. The results of the A_D1_/A_G_ ratios are presented in Table S1 (Supporting Information), revealing that the value for SAHTC‐1300 (1.95) is higher than those for SCG‐1300 (1.77) and HTC‐1300 (1.79). This indicates that SAHTC‐1300 possesses a higher degree of disorder.^[^ ^17^ ^]^ Integrating the findings from HRTEM, XRD, and Raman spectroscopy analyses, it is discerned that SAHTC exerts a modulating influence on the interlayer spacing, defect concentration, and structural order within the hard carbon materials.

In order to comprehensively understand the internal pore structure of HC materials, N_2_ adsorption‐desorption analyses were performed. The Brunauer‐Emmett‐Teller (BET) method revealed that HTC‐ and SAHTC‐pretreated hard carbons exhibit enhanced specific surface area and total pore volume compared to untreated counterparts (Figure S10a,b, Supporting Information). Barret‐Joyner‐Halenda (BJH) analysis of the desorption isotherm branches quantified the mesopore size distributions (Figure 2f). While SCG‐1300 and HTC‐1300 showed Type II isotherms with H_4_‐type hysteresis loops, SAHTC‐1300 displayed a distinct Type IV isotherm, suggesting its hierarchical micropores and mesopores structure. Complementary CO_2_ adsorption measurements (Figure 2g; Figure S10c, Supporting Information) specifically probed ultra‐micropores (<1 nm), revealing pore size distributions between 0.4 to 0.85 nm for all samples.^[^ ^18^ ^]^ Notably, HTC‐1300 and SAHTC‐1300 displayed pore volumes of 0.015 and 0.012 cm^3^ g^−1^, respectively, representing enhancements over SCG‐1300 (0.006 cm^3^ g^−1^). Ultra‐micropores have been shown to facilitate Na⁺ transport and promote electrolyte desolvation via nanoconfinement effects.^[^ ^19^ ^]^ Further detailed structure of closed pores in HC materials is elucidated by small‐angle X‐ray scattering (SAXS) and true density analyses based on Archimedes principle. SAXS patterns of HCs reveal a broad humps at the scattering vector Q of 1–2 nm^−1^ (Figure 2h), reflecting closed pores (micrometer and nanometer‐scale voids between sp^2^ graphitic layers) in the carbon matrix.^[^ ^20^ ^]^ Based on the spherical closed pore model, the characteristic length related to the variation in scattering intensities was fitted (Figure S11, Supporting Information), providing the average diameters of the closed pores decreasing from 2.5 nm for SCG‐1300 to 2.23 nm for HTC‐1300, and further to 2.08 nm for SAHTC‐1300. These results indicate that SAHTC‐1300 has both reduced average pore size and higher content of closed pores compared to the other two samples. True density analysis serves as a robust methodology for characterizing closed porosity in carbonaceous materials. This technique effectively eliminates interference from open pore networks and interparticle void spaces during measurements, thereby enabling precise determination of the combined volume occupied by closed pores and the solid matrix. Given that defect‐free graphite with an ideal layered structure (containing negligible closed porosity) exhibits a theoretical true density of 2.26 g cm^−3^, the closed pore volume of HC can be calculated using the following formula:^[^ ^21^ ^]^

(1) $$V_{closedpore}=\frac{1}{\rho_{ture}}-\frac{1}{2.26}$$

As shown in Figure 2i, SAHTC‐1300 exhibits the highest closed pore volume, which is consistent with the results obtained from small‐angle X‐ray scattering (SAXS) measurements. This observation demonstrates that the pretreatment process reduces the radius of closed pores within HC while simultaneously increasing their population density, thereby inducing an overall augmentation in closed pore volume and thus creating additional accommodation space for sodium storage.

**Figure 3:**
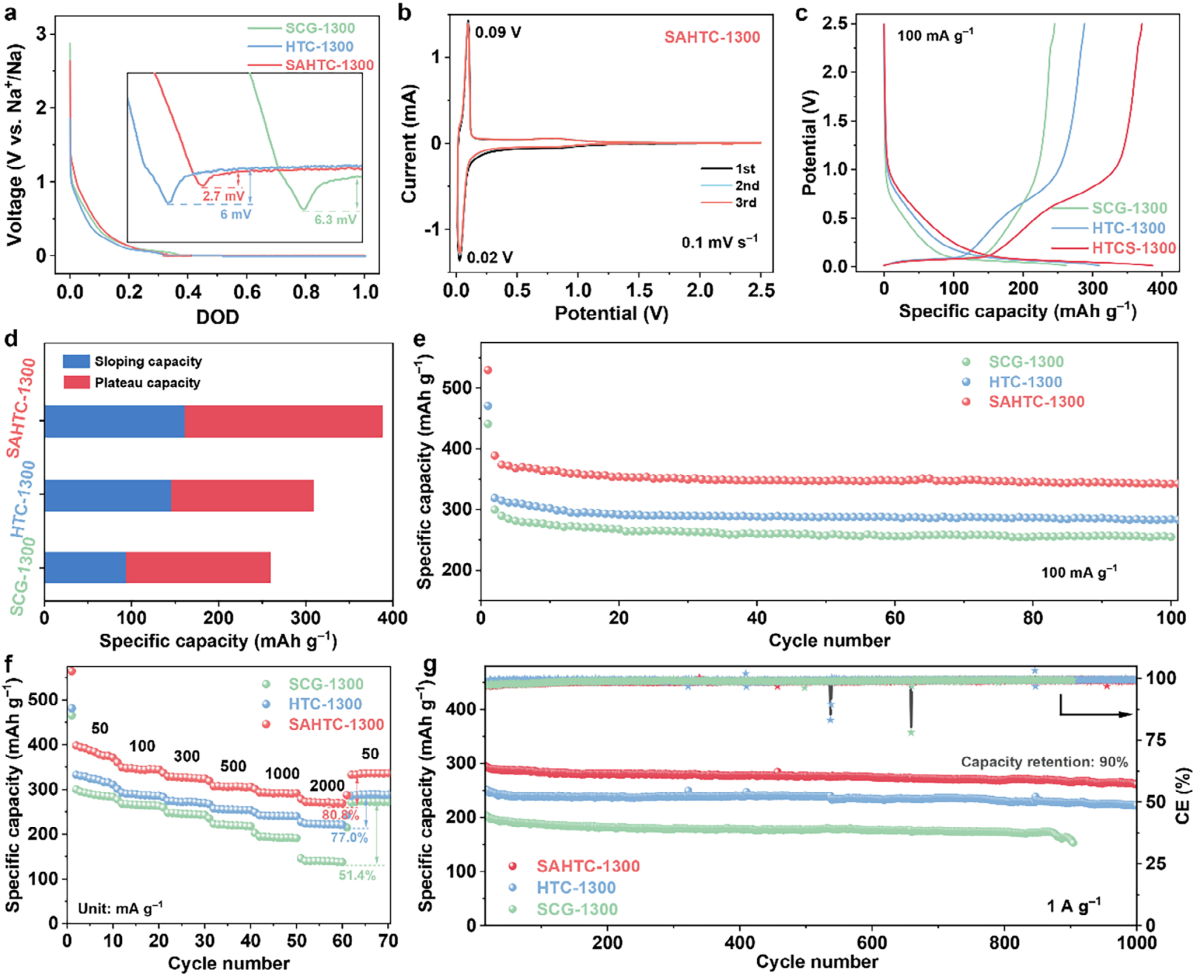
Electrochemical performance of SCG‐1300, HTC‐1300, and SAHTC‐1300 electrodes. a) The voltage‐time curves during Na nucleation at 0.08 mA cm^−2^ on electrode. b) CV curves of SAHTC‐1300 during the initial three cycles. c) Representative second‐cycle charge/discharge profiles. d) Comparative analysis of plateau and slope capacities derived from the second‐cycle charge/discharge characteristics at 50 mA g^−1^. e) Cyclic stability assessment at 100 mA g^−1^. f) Rate capability evaluation across current densities ranging from 50 mA g^−1^ to 2 A g^−1^. g) Long‐term cycling performance measured at 1 A g^−1^.

Electrochemical performance of synthesized HC anodes was systematically evaluated in half cell with metallic sodium as both counter and reference electrodes. As illustrated in **Figure** **3**a, nucleation overpotentials are employed to evaluate the degree of sodiophilicity on the closed pore interface quantitatively. The curve of SAHTC‐1300 electrode exhibits much more smooth voltage dip at the nucleation stage, with a nucleation overpotential of only 2.7 mV, which is much smaller than that of SCG‐1300 and HTC‐1300 electrode. Owing to the more sodiophilic oxygen‐containing functional groups (C═O) in SAHTC‐1300, the electrode exhibits lower resistance for metallic Na to plate at the pore interface.^[^ ^22^ ^]^ the charge/discharge curves of all three samples can be divided into a slope region (above 0.1 V) and a plateau region (below 0.1 V), indicating the typical Na‐ion storage behavior of hard carbon. The high‐potential plateau observed at 0.4–0.7 V is attributed to the interaction between Na⁺ and C═O groups.^[^ ^23^ ^]^ Notably, SAHTC‐1300 demonstrated superior electrochemical performance, delivering a reversible capacity of 386 mAh g^−1^ at 50 mA g^−1^, substantially exceeding those of SCG‐1300 (260 mAh g^−1^) and HTC‐1300 (307 mAh g^−1^). Comparative analysis of capacity contributions (Figure 3b) reveals distinct storage mechanism that HTC‐1300 predominantly relies on slope‐region capacity (145 mAh g^−1^, 47% of the total capacity), likely attributable to its maximized specific surface area and surface‐mediated group of Na⁺ adsorption. Conversely, SAHTC‐1300 achieves remarkable capacity enhancement through dominant plateau‐region contributions (220 mAh g^−1^, 61% of the total capacity), accounting for which is higher than that of SCG‐1300. The first‐cycle charge/discharge curves (Figure S12, Supporting Information) reveal that SAHTC‐1300 exhibits significantly enhanced capacities in both the plateau and slope regions compared to SCG‐1300. This improvement of SAHTC‐1300 electrode is ascribed to pore‐orifice nanoconfinement, abundant C═O functional groups, and proportion closed‐pore structure, which facilitate Na⁺ nucleation within the closed pores.^[^ ^24^ ^]^ Simultaneously, SAHTC‐1300 demonstrates the highest initial Coulombic efficiency (ICE) of 70%, significantly surpassing those of SCG‐1300 (56%) and HTC‐1300 (64%). This can be attributed to the lower specific surface area and fewer irreversible surface defects of SAHTC‐1300, while the abundant C═O groups facilitate the formation of a NaF‐rich solid electrolyte interphase (SEI) on the hard carbon surface (Figure S13, Supporting Information),^[^ ^25^ ^]^ enhancing interfacial stability and reducing irreversible capacity loss. All three samples demonstrated excellent cycling stability at 100 mA g^−1^ (Figure 3e). To elucidate the synergistic sodium storage mechanism between pores (pore‐orifice and closed pore) and C═O functional groups within both slope and plateau regions, its impact on rate capability can be further explored. As the current density increase from 50 to 2000 mA g^−1^, the SAHTC‐1300 and HTC‐1300 electrode exhibits a capacity retention of 80.8% and 77.0% at a high‐rate of 2000 mA g^−1^, respectively (Figure 3f; Figure S14, Supporting Information). Crucially, SCG‐1300 electrode exhibits the lowest specific capacity at current densities (50–2000 mA g^−1^) and suffers significant capacity degradation at high rates, retaining only 51.4% under 2000 mA g^−1^ (140 mAh g^−1^). This discrepancy stems from the increased carbonyl groups and pore structures in SAHTC‐1300, which induce extended sloping and plateau regions (Figure S15, Supporting Information), thereby simultaneously enhancing both sodium storage capacity and kinetics. Remarkably, SAHTC‐1300 maintained a high reversible capacity of 262 mAh g^−1^ after 1000 cycles at 1 A g^−1^ (Figure 3g), corresponding to an outstanding capacity retention of 90%.

**Figure 4:**
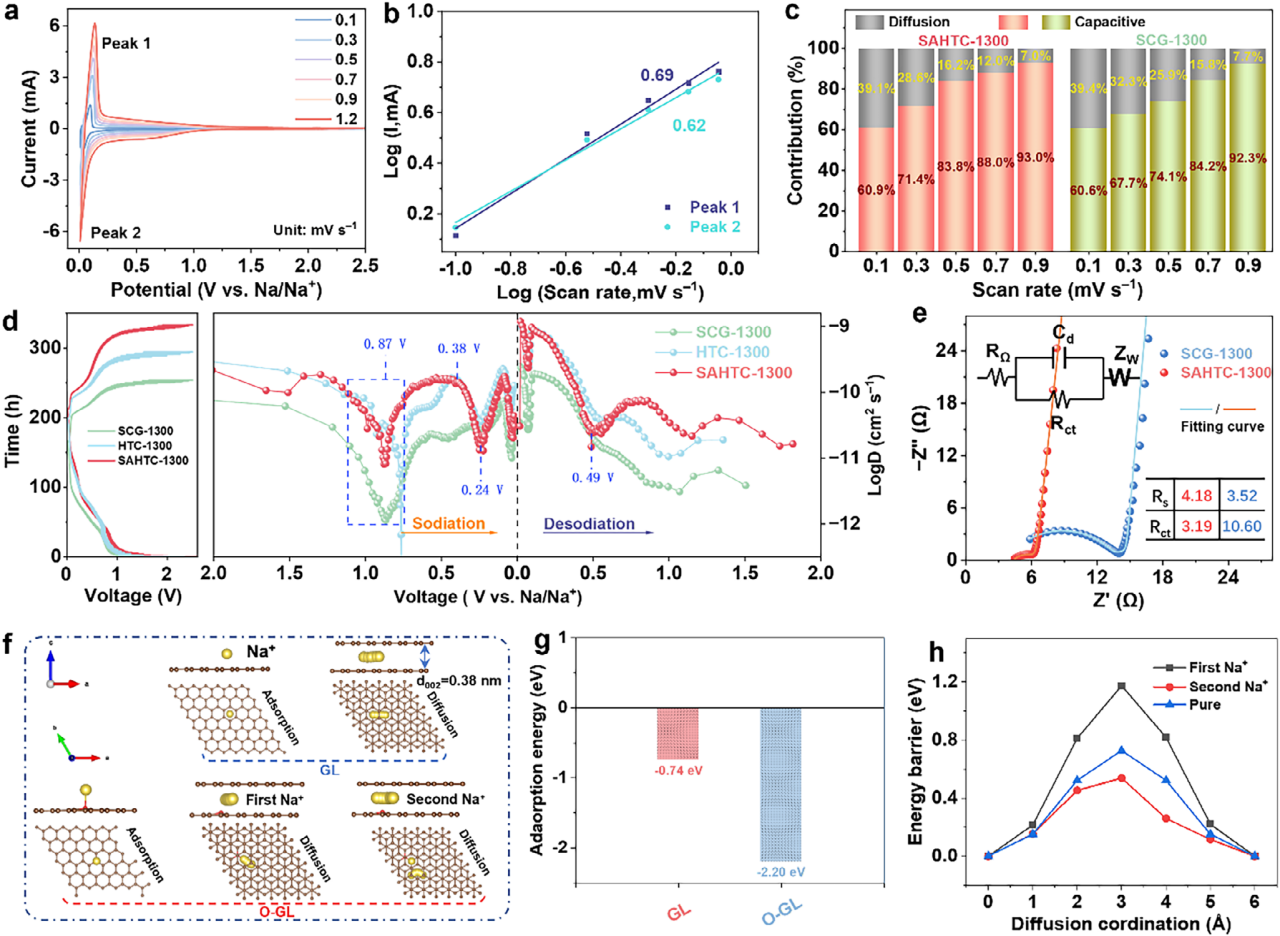
a) Multi‐rate scanning profiles of SAHTC‐1300. b) Diffusion and capacitive contributions at various scan rates of SAHTC‐1300. c) Linear relationships of log (i) versus log (v) for the peaks at 0.01 and 0.3 V of SAHTC‐1300. d) GITT curves of SCG‐1300, HTC‐1300, and SAHTC‐1300, along with the calculated Na⁺ diffusion coefficients (D_Na⁺_) during the resting and desodiation processes. e) Nyquist plots of SCG‐1300 and SAHTC‐1300 (inset is the model of an equivalent circuit). f) Schematic of Na^+^ adsorption sites (monolayer) and Na^+^ diffusion paths (bilayer) between GL and O‐GL. Adsorption energy g) and diffusion barrier energies h) of Na^+^ in different models.

In order to explore the electrochemical kinetics and gain insight into the Na^+^ storage process, CV measurements were performed at scan rates ranging from 0.1 to 1.2 mV s^−1^ (**Figure** **4**a; Figure S16, Supporting Information). The CV curves display similar peaks at various scan rates, exhibiting the low polarization and excellent reversibility. Additionally, the contributions of capacitive‐controlled and diffusion‐controlled processes were analyzed using the CV measurement method. To qualitatively assess the level of capacitive effects, the relationship between the natural logarithm of the scan rate ln *v* and the natural logarithm of the peak current ln *i* was plotted according to the following formula:^[^ ^26^ ^]^

(2) $$i=av^{b}$$

Here, the value of *b *represents the slope of the ln *i* versus ln *v* curve. Specifically, when the value of *b* approaches 0.5, it indicates a diffusion‐controlled process, whereas a value of *b* close to 1 suggests capacitive behavior.^[^ ^27^ ^]^ For SAHTC‐1300, the *b* values were determined to be 0.69 and 0.62 (Figure 4b), indicating that its sodium storage behavior is governed by a combination of diffusion‐controlled and capacitive processes. Based on previous studies, the proportion of capacitive contribution can be further quantified. At a specific potential *V*, the current response *i* can be deconvoluted into contributions from surface capacitive behavior and diffusion‐controlled processes, as described by the following relationship:^[^ ^28^ ^]^

(3) $$i\left(V\right)=k_{1}v+k_{2}v^{1/2}$$

The surface capacitive behavior can be described as *k*_1_ *ν*, while the diffusion‐controlled process can be expressed as *k*_2_ *ν* ^1/2^. Therefore, by determining the values of *k*_1_ *ν* and *k*_2_ *ν* ^1/2^, the fractional contribution of each mechanism to the total current at a specific potential can be quantified. As shown in Figure 4c, the capacitive contribution of SAHTC‐1300 increases from 69.88% at 0.1 mV s^−1^ to 93% at 1.2 mV s^−1^. This increase in capacitive contribution demonstrates that the capacitive process plays a critical role in electrochemical storage, particularly at high current densities. These results highlight the importance of capacitive behavior in enhancing the rate capability and overall performance of sodium ion storage systems.

Furthermore, the galvanostatic intermittent titration technique (GITT) was employed to analyze the three electrodes under a pulse current of 20 mA g^−1^. The sodium ion diffusion coefficients (*D*_Na_^+^) were calculated during the sodiation and desodiation processes.^[^ ^29^ ^]^ Notably, the similar GITT profiles suggest analogous sodium storage mechanisms in both hard carbon materials (Figure 4d). The diffusion coefficient initially increases and then decreases at ≈0.56 V, which can be attributed to the formation of the solid electrolyte interphase (SEI) layer with good structural stability (Figure S17, Supporting Information). Subsequently, the diffusion coefficient exhibits typical rising and falling trends due to the Na^+^ adsorption process and interlayer insertion process (0.38–0.24 V). The increase in the diffusion coefficient within the range of 0.04–0 V indicates the presence of a typical pore‐filling process. Moreover, compared to SCG‐1300 and HTC‐1300, SAHTC‐1300 demonstrates a remarkable enhancement in *D*_Na_^+^ values throughout the entire process, suggesting that the pretreatment facilitates the construction of an advanced porous structure. This may explain its unprecedented rate performance and rapid Na^+^ diffusion. Additionally, based on electrochemical impedance spectroscopy (EIS) measurements (Figure 4e), the SAHTC‐1300 electrode exhibits a lower charge transfer resistance (*R*_ct_) both before cycling and after ten cycles. To further investigate the underlying mechanisms for the correlations between the structure and the sodium storage properties of the SAHTC‐1300 material, Density Functional Theory (DFT) calculations were employed to elucidate how C═O functional groups enhances its kinetic performance. As depicted in Figure 4f, DFT calculations based on graphene model reveal that C═O improves sodium storage performance by simultaneously modifying thermodynamic properties and diffusion pathways. Specifically, in the monolayer graphene adsorption model, C═O reduces the Na⁺ adsorption energy from ‐0.74 eV (GL) to ‐0.79 eV (O‐GL), enabling faster intercalation (Figure 4g). Bilayer graphene diffusion analysis (Figure 4h) reveals that initial Na⁺ storage at oxygen sites requires overcoming a higher energy barrier (1.17 eV vs. 0.73 eV in GL), which stabilizes trapped ions. Subsequent Na⁺ diffusion barriers, however, decrease significantly to 0.53 eV (Figure 4h). This dual effect with preferential storage at oxygen sites followed by low barrier diffusion accelerates Na^+^ transport kinetics in the low potential region, ultimately enhancing sodium storage performance of SAHTC‐1300. In summary, introducing more C═O groups along with optimally sized pore structures (including tailored pore orifices and closed pores) facilitates rapid electron/Na^+^ transport while reducing nucleation energy barriers at pore edges, thereby enhancing reaction kinetics for efficient sodium storage.

**Figure 5:**
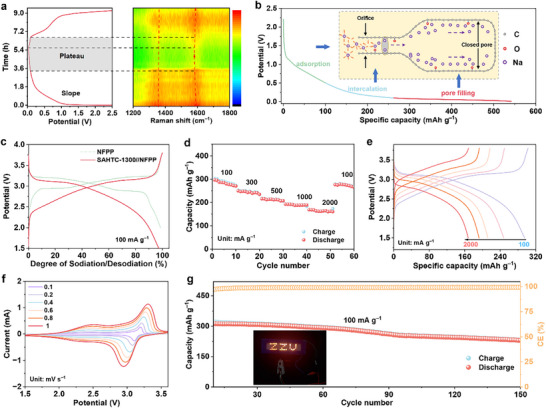
a) In situ Raman spectra of SAHTC‐1300. b) Sodium storage mechanisms at different discharge stages based on a simplified closed pore structure model. c) Charge–discharge profiles of the Na_4_Fe_3_(PO_4_)_2_P_2_O_7_ (NFPP) cathode and SAHTC‐1300//NFFP full cell. The electrochemical performance of SAHTC‐1300//NFPP: d) Rate capability evaluated under varying current densities, e) charge/discharge curves measured at different current densities, f) cyclic voltammetry (CV) curves obtained at multiple scan rates, and g) cycling performance at 100 mA g^−1^.

The structural evolution of SAHTC‐1300 during the charge‐discharge process was investigated using in situ Raman spectroscopy and ex situ X‐ray photoelectron spectroscopy. As shown in **Figure** **5**a, during the discharge process, the intensity of the two broad peaks gradually decreases in the high‐voltage region, reaches its minimum in the medium‐voltage region, and then remains stable in the low‐voltage region. This phenomenon can be attributed to the suppression of the A_1g_ symmetric (D‐band) breathing mode and the E_2g_ symmetric (G‐band) in‐plane bond stretching vibrations of carbonaceous materials, caused by both the adsorption of Na⁺ on the surface and the storage of Na⁺ within the bulk.^[^ ^30^ ^]^ Considering that surface reactions have a weaker structural modification capability compared to bulk reactions, the sodium storage behavior of SAHTC‐1300 in the high‐voltage and medium‐voltage regions is likely dominated by capacitive behavior and Na⁺ intercalation, respectively. In the low‐voltage region, the sodium storage behavior may correspond to the filling of sodium within the nanopores in the plateau region. This interpretation is supported by the quasi‐metallic sodium peak shift observed in ex situ XPS (Figure S18, Supporting Information). Electrodes at different discharge states were immersed in an ethanolic phenolphthalein solution (Figure S19, Supporting Information), with the solution exhibiting the most pronounced coloration at the fully discharged state (0.01 V), suggesting the highest concentration of quasi‐metallic sodium species at this potential.^[^ ^31^ ^]^ According to the TEM images of the SAHTC‐1300 electrode discharged to 0.01 V (Figure S20, Supporting Information), the closed pores are fully filled with quasi‐metallic sodium. Based on this analysis, the proposed Na^+^ ion storage mechanism for biomass‐based hard carbon of SAHTC‐1300 is schematically presented in Figure 5b with three sequential phases: stage I (2.5‐0.38 V), characterized by Na^+^ adsorption on the surface or defect sites; stage II (0.38‐0.24 V), involving desolvation and intercalation process; Stage III (0.24‐0.01 V), where Na^+^ occupies the pore spaces.

For practical applications, a sodium‐ion full cell was fabricated by pairing SAHTC‐1300 as the anode with NFPP as the cathode (Figure 5c). The SAHTC‐1300//NFPP full cell delivered a high reversible capacity of 311.95 mAh g^−1^ at a current density of 100 mA g^−1^, with an output voltage of 3.2 V. The rate performance of the assembled battery was tested and provided in Figure 5d, high discharge capacities of 286.5, 247.2, 213.2, 191.4, and 163.7 mAh g^−1^ can be obtained at 100, 300, 500, 1000, and 2000 mA g^−1^, suggesting its superior rate performance. The charge‐discharge profiles and cyclic voltammetry (CV) curves of the full cell indicated good reversibility (Figure 5e,f). Moreover, the full cell exhibits excellent cycling stability at room temperature, retaining ≈73.7% capacity after 150 cycles at a current density of 100 mA g^−1^ (Figure 5g). The inset photograph in Figure 5d further confirm the practical potential of SAHTC‐1300//NFPP full cell by illuminating the “ZZU” patterned light‐emitting diodes (LED).

## 3 Conclusion

In summary, this study demonstrates the influence of sulfuric acid‐assisted hydrothermal pretreatment on the structural characteristics and electrochemical sodium storage performance of biomass‐derived hard carbon. The sulfuric acid‐mediated hydrothermal strategy facilitates the formation of a crosslinked small‐molecule structure in the precursor material. The resulting hard carbon exhibits optimally sized pore structures and the introduction of C═O functional groups with reduced nucleation energy barrier. These structural modifications endow the obtained hard carbon with exceptional reversible specific capacity and remarkable fast‐charging capability. The results indicate that the SAHTC‐1300 hard carbon anode outperforms both HTC‐1300 and SCG‐1300, delivering the highest reversible specific capacity (386 mAh g^−1^ at 50 mA g^−1^), superior rate capability (270 mAh g^−1^ at 2 A g^−1^), and outstanding cycling stability (90% capacity retention after 1000 cycles at 1 A g^−1^). This work elucidates the feasibility of sulfuric acid‐assisted hydrothermal carbonization for the synthesis of high‐performance biomass‐derived hard carbon anodes for sodium‐ion batteries, providing critical insights for future development of other biomass‐based hard carbon materials.

## Conflict of Interest

The authors declare no conflict of interest.

## Supporting information



Supporting Information

## Data Availability

The data that support the findings of this study are available in the supplementary material of this article.
