# Supplementary material for: Regulating Pores and Carbonyl Groups of Biomass‐Derived Hard Carbon for Enhanced Sodium Storage
Source: Adv Sci (Weinh). 2025 Jul 30;12(40):e10328. doi: 10.1002/advs.202510328 (PMC12561183; doi:10.1002/advs.202510328)
Supplement: Supplementary file 1 — Supporting Information [file ADVS-12-e10328-s001.docx]

**Supporting Information**

**Regulating Pores and Carbonyl Groups of Biomass-Derived Hard Carbon for Enhanced Sodium Storage**

*Chi Chen^1,†^, Yapeng Tian^1,†^, Run Ren^2^,* *Shuocong Duan^1^, Dandan Wang^1^, Zhuosen Wang^1^, Yunfeng* *Chao^1,^*, Jianhua Zhu^1,^*, Xinwei Cui^1,^**

*^1^Henan Institute of Advanced Technology, Zhengzhou University, Zhengzhou 450003, People’s Republic of China*

*^2^ Zhongyuan Critical Metals Laboratory, Zhengzhou University, Zhengzhou 450001, China*

^†^ These authors contribute equally to this work.

**E-mail addresses:* jianhuazhu@zzu.edu.cn (J. H. Zhu); chaoyf@zzu.edu.cn (Y. F. Chao); xinweic@zzu.edu.cn (X. W. Cui).

**Experimental Section**

**Synthesis of hard carbons**

The detailed preparation procedure is illustrated in Figure S1. Initially, spent coffee grounds (SCG) were washed with deionized water and dried for 12 hours. For the hydrothermal treatment, 10 g of SCG was dispersed in 100 g of 0.1 M H_2_SO_4_ solution under continuous magnetic stirring, and the resulting precursor (ASCG) solution was transferred into a polyphenol-lined autoclave. The autoclave was subsequently sealed with a stainless-steel jacket and heated at 200 °C for 2 h. After cooling, the obtained precipitate was thoroughly washed with deionized water and ethanol, followed by drying at 80 °C overnight to yield the solid product denoted as SAHTC. For comparative purposes, a control sample (HTC) was prepared using an identical hydrothermal process without the addition of H_2_SO_4_. Finally, the samples were subjected to thermal treatment at 1300 °C for 2 h under an argon atmosphere with a heating rate of 5 °C/min, producing the corresponding carbonized materials designated as SCG-1300, HTC-1300, and SAHTC-1300, respectively.

**Materials characterization**

The surface morphology of the synthesized product was characterized by scanning electron microscopy (SEM, ZEISS Gemini 300). The internal structure and morphology of the obtained samples were examined using transmission electron microscopy (TEM, FEI Tecnai F20/TF30) and high-resolution transmission electron microscopy (HRTEM). Crystalline structure analysis was performed by powder X-ray diffraction (XRD, PANalytical Empyrean) with Cu Kα radiation (λ = 1.5418 Å). Raman spectroscopic measurements were conducted using a LabRAM Soleil nano Raman spectrometer to identify vibrational modes and determine crystal symmetry. CO_2_ adsorption-desorption isotherms and pore size distribution measurements were obtained at 273 K using an ASAP2020 analyzer. X-ray photoelectron spectroscopy (XPS) analysis was performed on a Thermo Scientific K-Alpha spectrometer to determine elemental valence states. Fourier transform infrared (FTIR) spectra were recorded on a Nicolet iS50 spectrometer. Thermogravimetric analysis (TGA) was carried out using a 1500DSP-SP instrument to quantify carbon content. True density measurements were conducted via helium pycnometry using an AccuPyc 1345 instrument to determine closed pore volume. Small-angle X-ray scattering (SAXS) data were collected at the 1W2A beamline of the Beijing Synchrotron Radiation Facility using a Cu Kα radiation source.

**Electrochemical measurements**

The electrochemical measurements were conducted by assembling CR2032-type coin cells in an argon-filled glove box. A homogeneous slurry was prepared by mixing 80 wt.% anode material (hard carbon, HC), 10 wt.% conductive additive (Super P), and 10 wt.% binder (polyvinylidene fluoride, PVDF) with an appropriate amount of N-methyl-2-pyrrolidone (NMP). The resulting slurry was cast onto copper foil, dried at 80°C in a vacuum oven for 12 h, and then punched into 12 mm diameter disks for cell assembly. Glass fiber separators (GF/D, Whatman) with a diameter of 19 mm were employed. Sodium metal foil was directly used as the counter electrode, and the electrolyte consisted of 1 M NaPF_6_ in diethylene glycol dimethyl ether (DEGDME). The typical mass loading of the electrodes ranged from 1 to 1.3 mg cm^–2^.

Galvanostatic charge/discharge measurements and galvanostatic intermittent titration technique (GITT) tests were performed on a LAND CT2001A battery testing system within a voltage window of 0.01–2.5 V (vs. Na^+^/Na). Rate capability was evaluated at sequential current densities of 50, 100, 300, 500, 1000, and 2000 mA g^–1^, while cycling performance was assessed at specific currents of 100 and 1000 mA g^–1^. Multi-rate cyclic voltammetry (CV, 0.01–2.5 V, 0.1–1.2 mV s^–1^) and electrochemical impedance spectroscopy (EIS) measurements were carried out using a Solartron multichannel electrochemical workstation.

For EIS characterization, coin cells identical to those used for galvanostatic testing (including identical electrolyte composition) were allowed to stabilize for 12 h at 25°C under open-circuit voltage conditions prior to measurement. A sinusoidal potential perturbation with 5 mV amplitude was applied across a frequency range of 1.0 MHz to 0.01 Hz. The acquired EIS data were subsequently analyzed and fitted using ZView software to determine key parameters including charge transfer resistance (R_ct_) and sodium ion diffusion impedance (Z_w_).

For cathode fabrication, a uniform slurry was prepared by mixing 80 wt.% cathode active material (Na_4_Fe_3_(PO_4_)_2_P_2_O_7_, NFPP), 10 wt.% Super P, and 10 wt.% PVDF in NMP, which was then coated onto carbon-coated aluminum foil, dried under identical conditions, and punched into 12 mm disks. The full cells were assembled using pre-sodiated hard carbon anodes, NFPP cathodes, and GF/D separators, with identical electrolyte composition to the half-cells. Electrochemical evaluation of full cells was performed on the LAND system within 1.5–3.8 V, with cycling tests at 100 mA g^–1^ and rate capability tests at progressively increasing current densities (100, 300, 500, 1000, and 2000 mA g^–1^).

**Computational methods**

Density functional theory (DFT) calculations were performed using the Vienna Ab initio Simulation Program (VASP), with the generalized gradient approximation (GGA) Perdew–Burke–Ernzerhof (PBE) functional to describe electron exchange and correlation. The projector-augmented plane wave (PAW) potentials were used to describe the core-valence electron interaction and take valence electrons into account using a plane wave basis set with a kinetic energy cutoff of 400 eV. Partial occupancies of the Kohn−Sham orbitals were allowed using the Gaussian smearing method and a width of 0.05 eV. The convergence criteria of structure optimization were chosen to be the maximum force on each atom less than 0.01 eV/Å with an energy change less than 1 × 10^−6^ eV. A k-points sampling of 3 × 3 × 1 with Monkhorst-Pack scheme was used in all calculations and all calculations were considered the spin polarization effect. The diffusion of Na on the as-established structures was studied using the CI-NEB method.


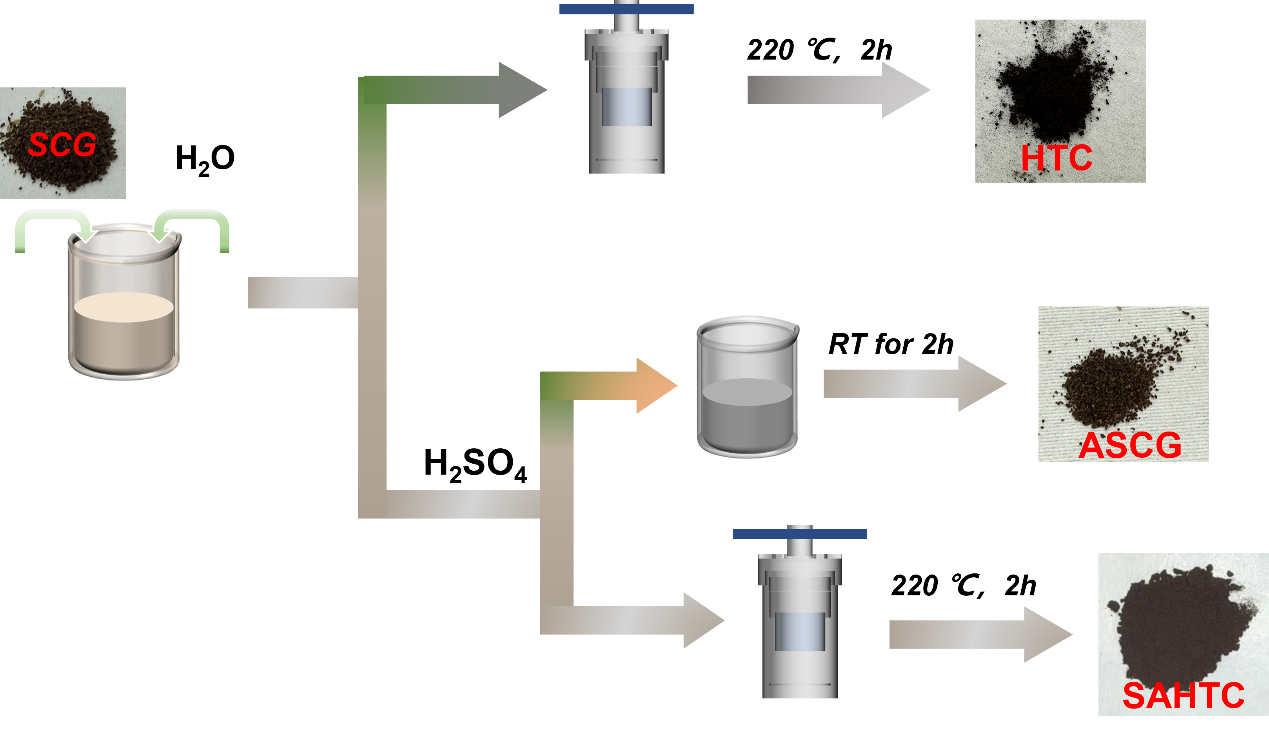


**Figure S1.** The flowchart for the synthesis of HTC, ASCG, and SAHTC.


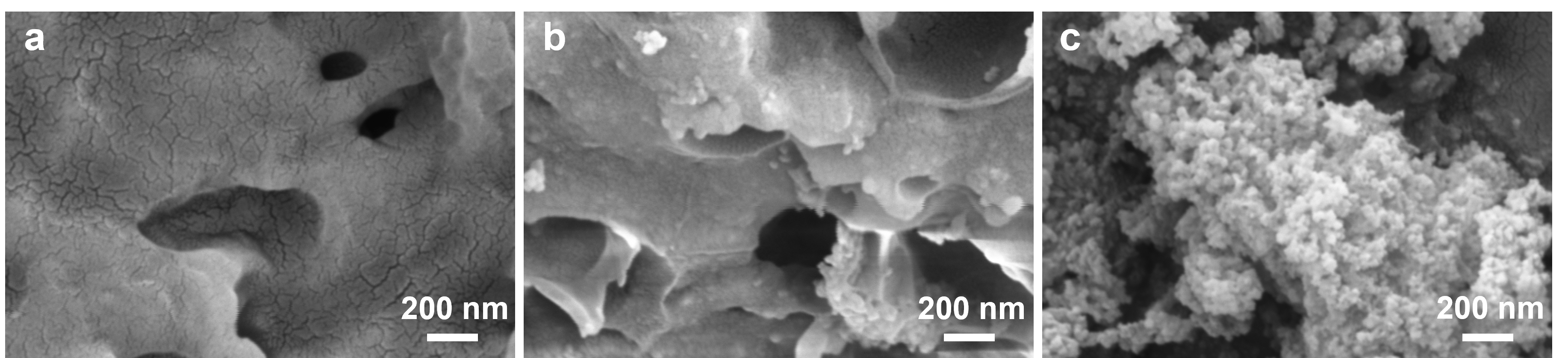


**Figure S2.** The SEM (Scanning Electron Microscopy) images of (a) SCG, (b) HTC, and (c) SAHTC.


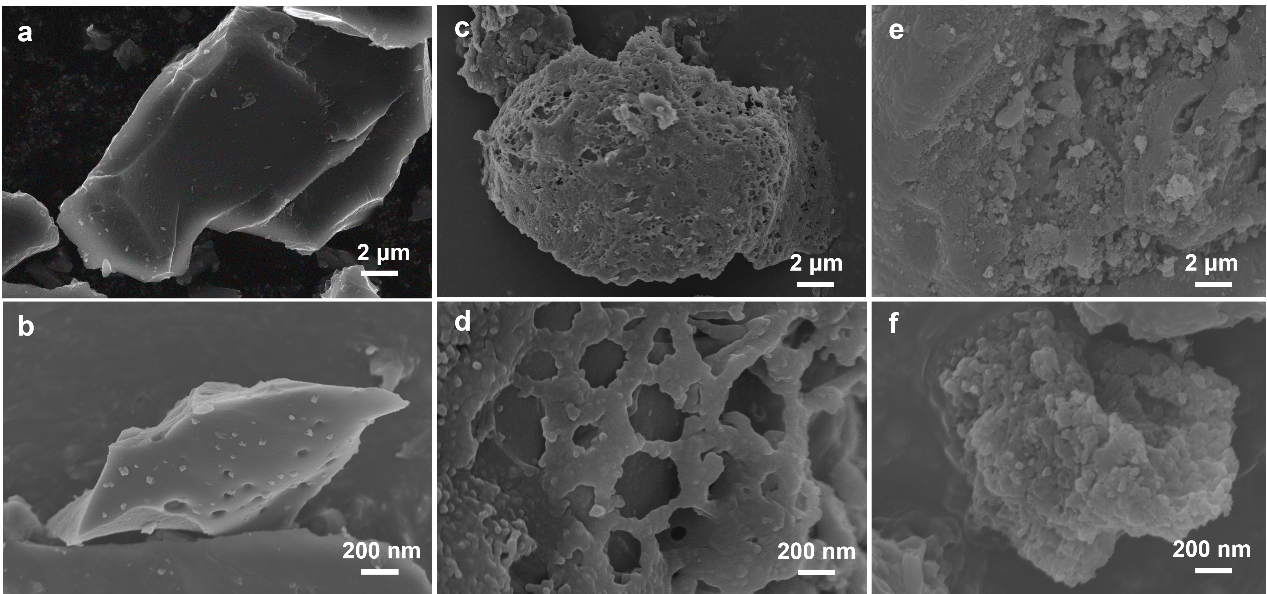


**Figure S3.** The SEM (Scanning Electron Microscopy) images of (a-b) SCG-1300, (c-d) HTC-1300, and (e-f) SAHTC-1300.


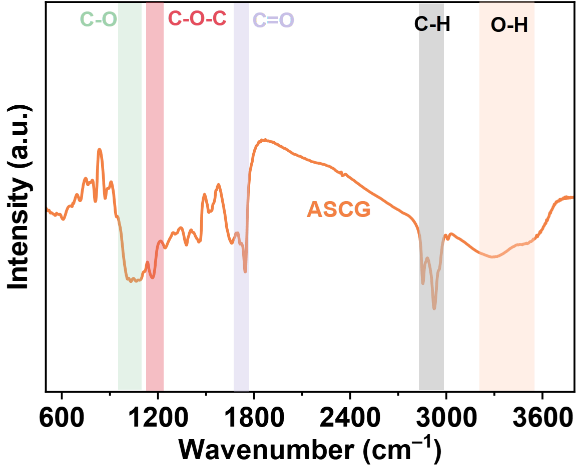


Figure S4. FTIR spectra of ASCG.


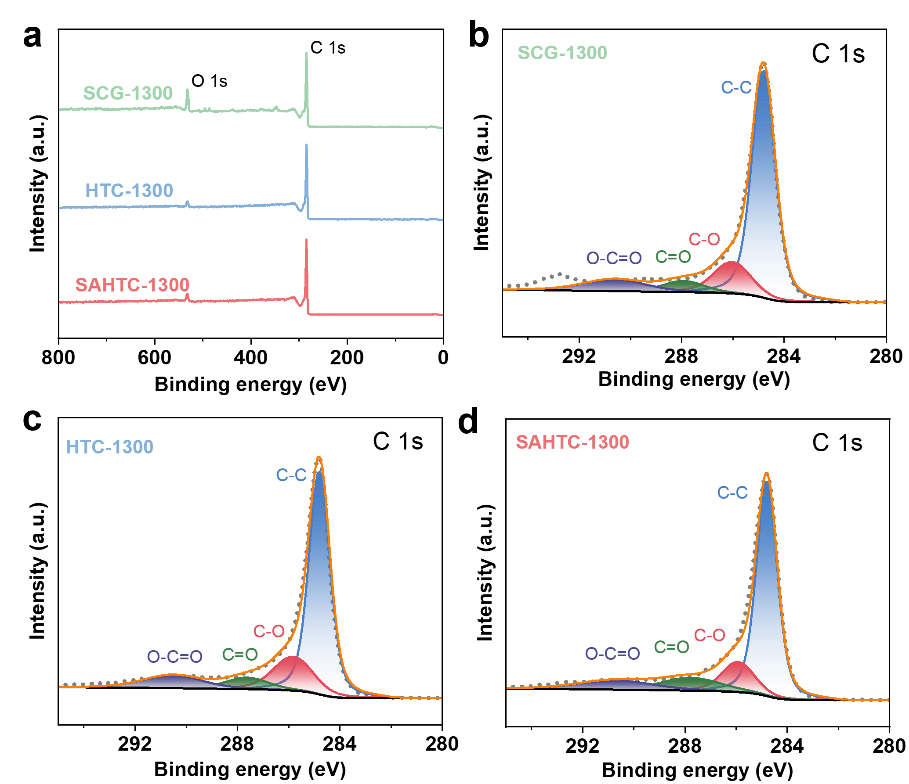


**Figure S5.** (a) The XPS survey spectra of SAHTC-1300, HTC-1300, and SCG-1300 High-resolution C1s XPS spectra of (b) SCG-1300, (c) HTC-1300, and (d) SAHTC-1300.

**
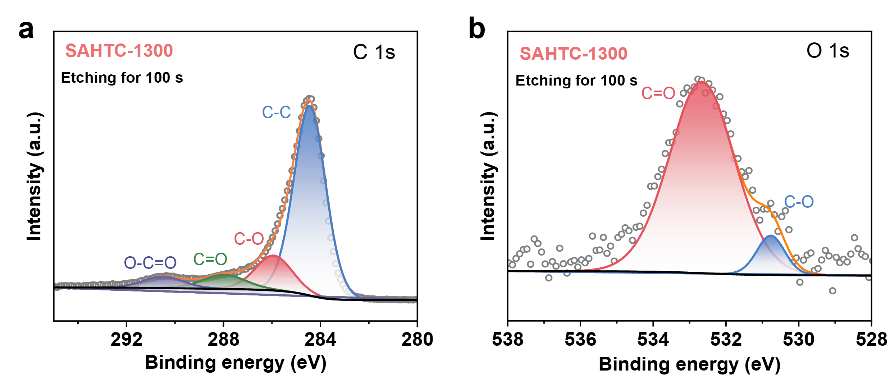
**

**Figure S6**. The (a) C1s and (b) O1s XPS spectra of SAHTC-1300 after 100 s of surface etching.


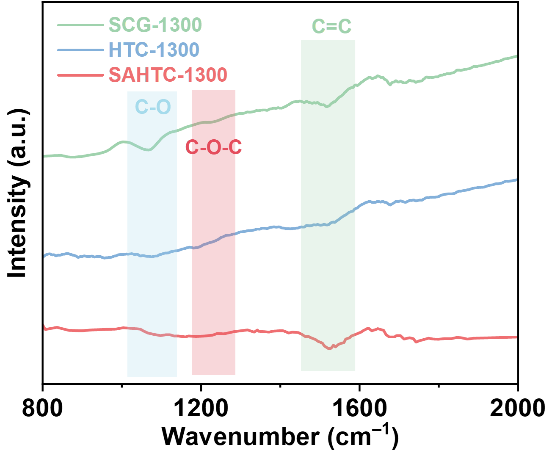


**Figure S7.** The infrared spectra of SAHTC-1300, HTC-1300, and SCG-1300.


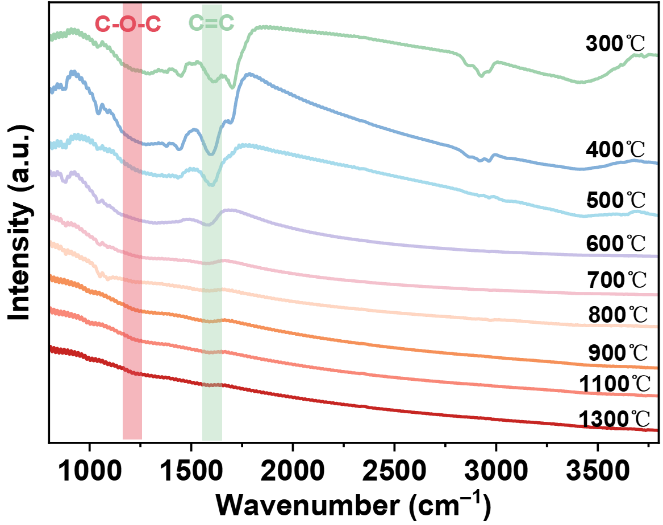


**Figure S8.** The *ex-situ* infrared spectroscopy of SAHTC-1300 at varying temperatures.

**Table S1.** The interlayer spacing, A_D1_/A_G_ ratio, and L_a_ values of SAHTC-1300, HTC-1300, and SCG-1300.

| Sample | d_(002)_  nm | A_D1_/A_G_  Raman | L_a_ (Raman)  nm |
| --- | --- | --- | --- |
| SCG-1300 | 0.365 | 1.77 | 10.86 |
| HTC-1300 | 0.371 | 1.79 | 10.74 |
| SAHTC-1300 | 0.384 | 1.95 | 9.85 |


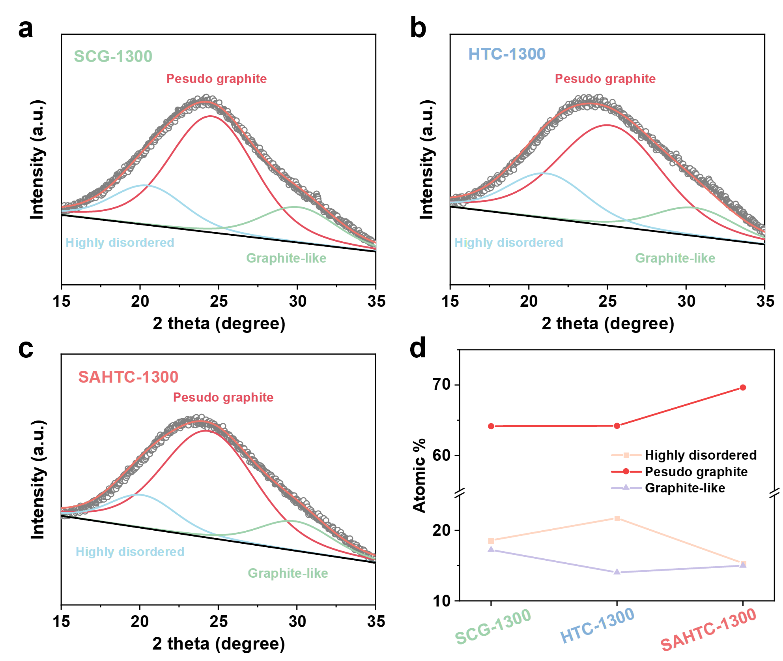


**Figure S9.** XRD pattern fitting curves of (a) SCG-1300, (b) HTC-1300, and (c) SAHTC-1300; (d) Percentage distribution of the three phases.


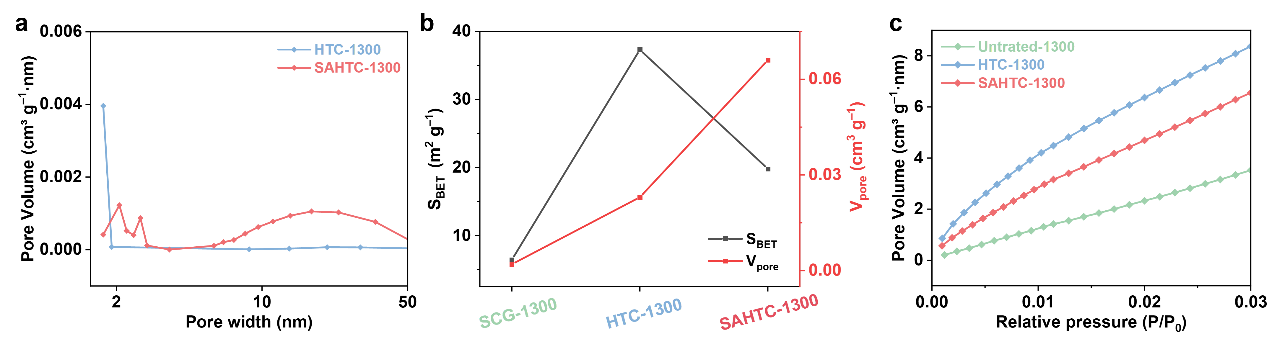


**Figure S10.** (a) Nitrogen adsorption-desorption pore size distribution, (b) specific surface area and pore volume, and (c) carbon dioxide adsorption-desorption isotherms of SAHTC-1300, HTC-1300, and SCG-1300.


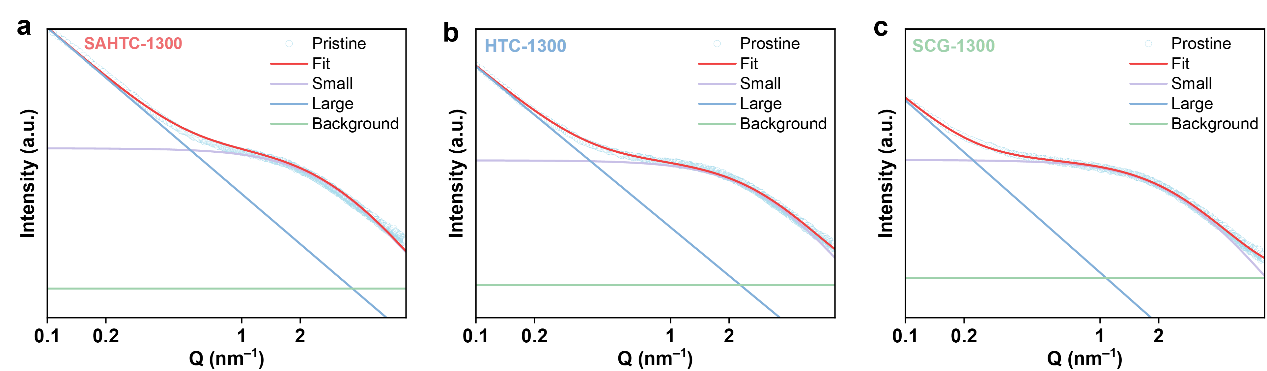


**Figure S11.** The SAXS fitting curves of (a) SAHTC-1300, (b) HTC-1300, and (c) SCG-1300.


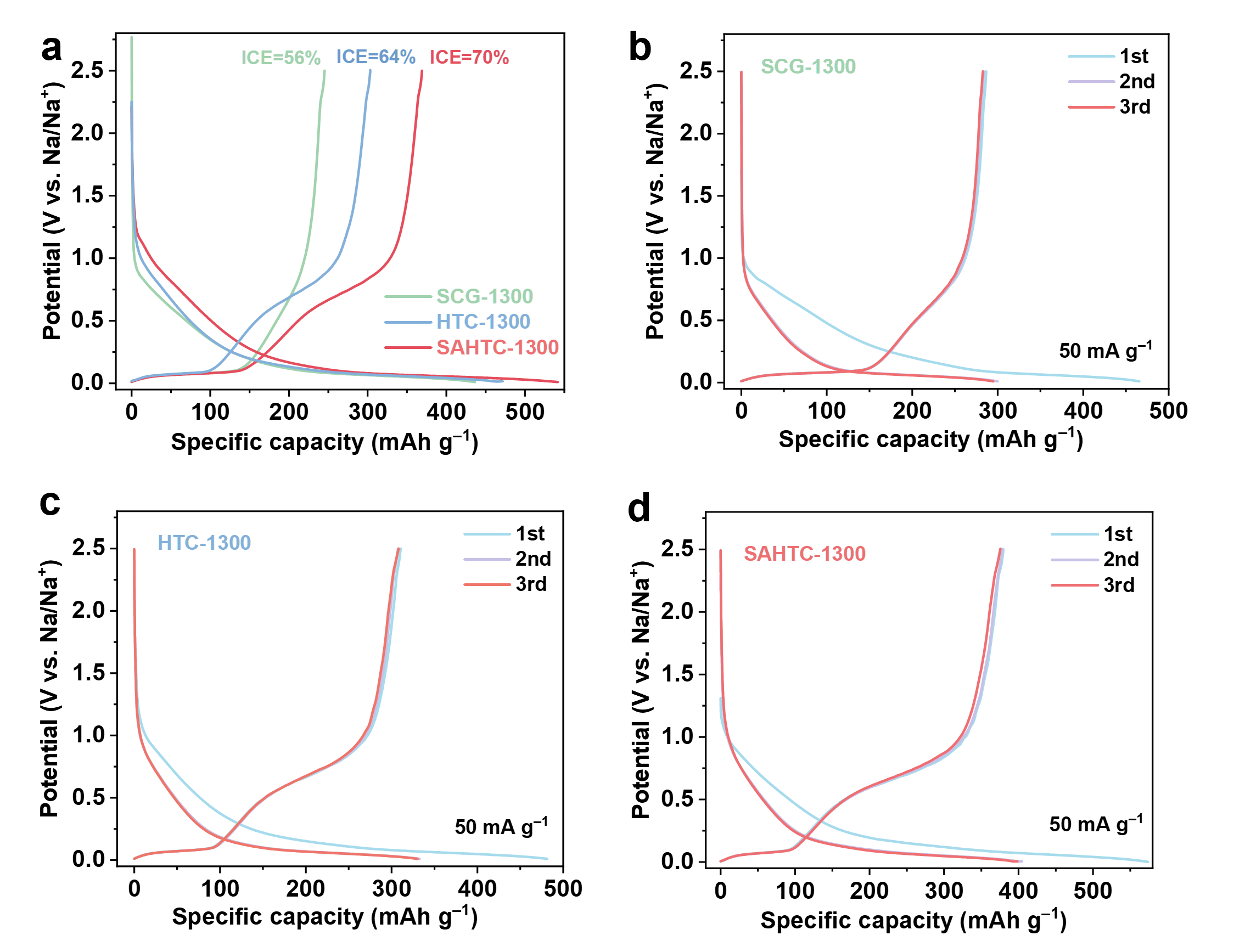


**Figure S12.** Initial charge-discharge profile of SCG-1300, HTC-1300, and SAHTC-1300. The charge/discharge profiles at 50 mA g^–1^ of (b) SCG-1300, (c) HTC-1300, and (d) SAHTC-1300.


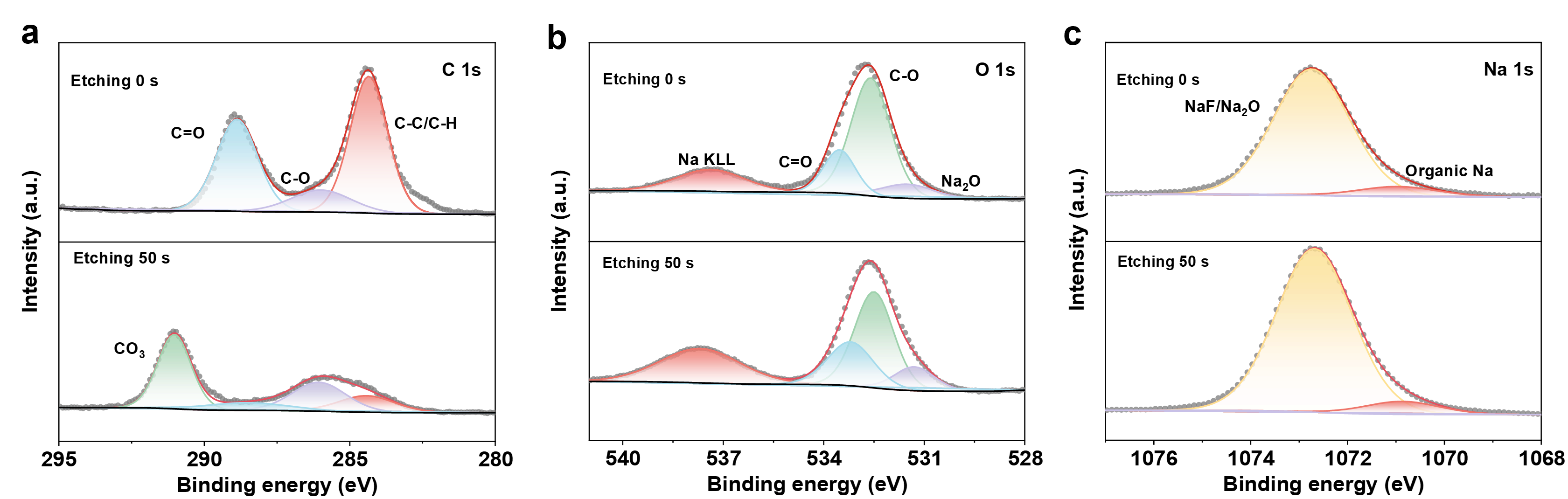


**Figure S13.** XPS spectra of SAHTC-1300 after full discharge: (a) C 1s, (b) O 1s, and (c) Na 1s.


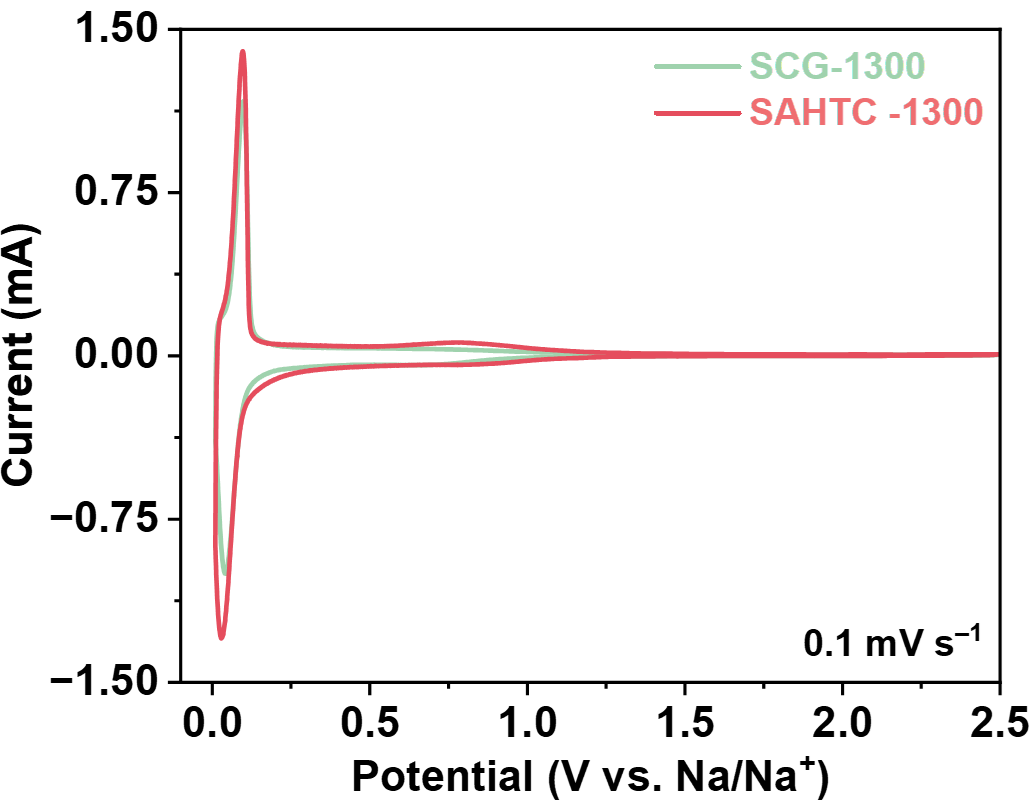


**Figure S14.** CV curves of SAHTC-1300 and SCG-1300 at a scan rate of 0.1 mV s^–1^.


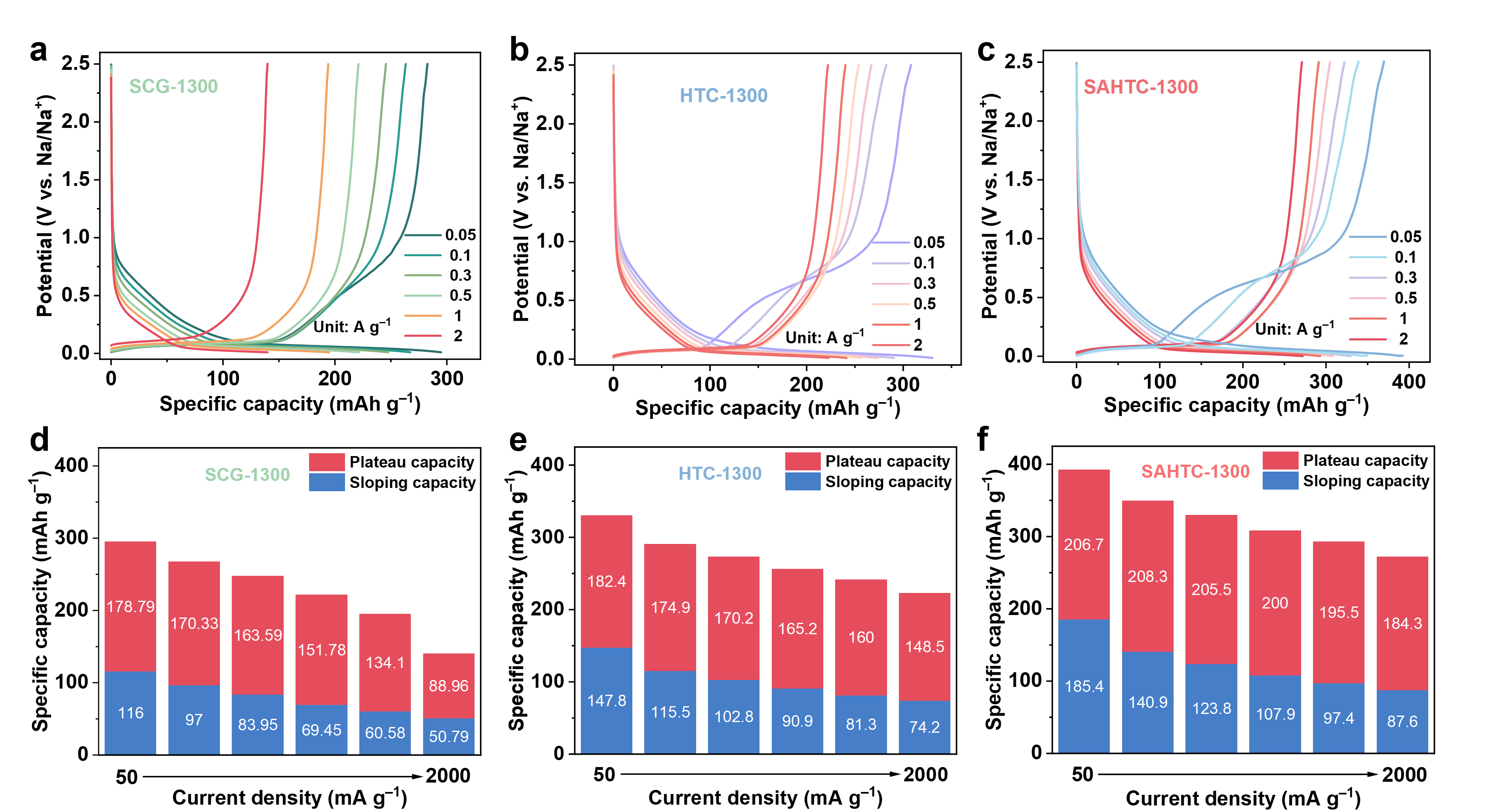


**Figure S15.** Galvanostatic charge/discharge profiles of (a) SCG-1300, (b) HTC-1300, and (c) SAHTC-1300 at various current densities. (c) Galvanostatic charge/discharge profiles of SCG-1300 at various current densities. Comparative analysis of plateau and slope capacities for (d) SCG-1300, (e) HTC-1300, and (f) SAHTC-1300 derived from different current densities.


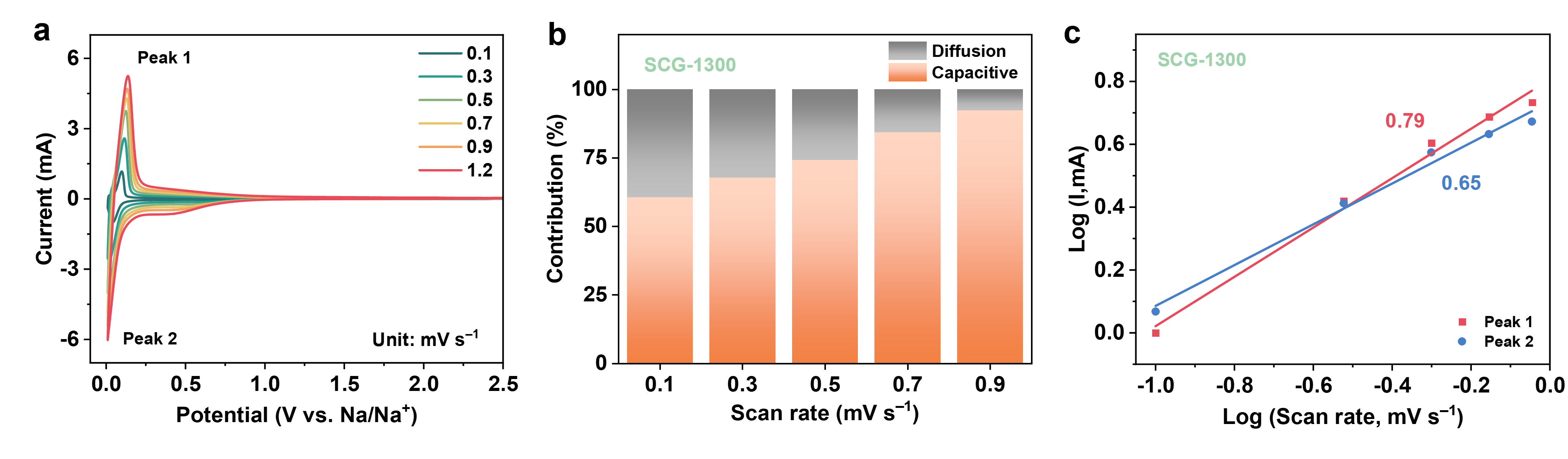


**Figure S16.** (a) Multi-rate scanning profiles of SCG-1300. (b) Diffusion and capacitive contributions at various scan rates of SCG-1300. (c) Linear relationships of log(i) versus log(v) for the peaks at 0.01 V and 0.3 V of SCG-1300.


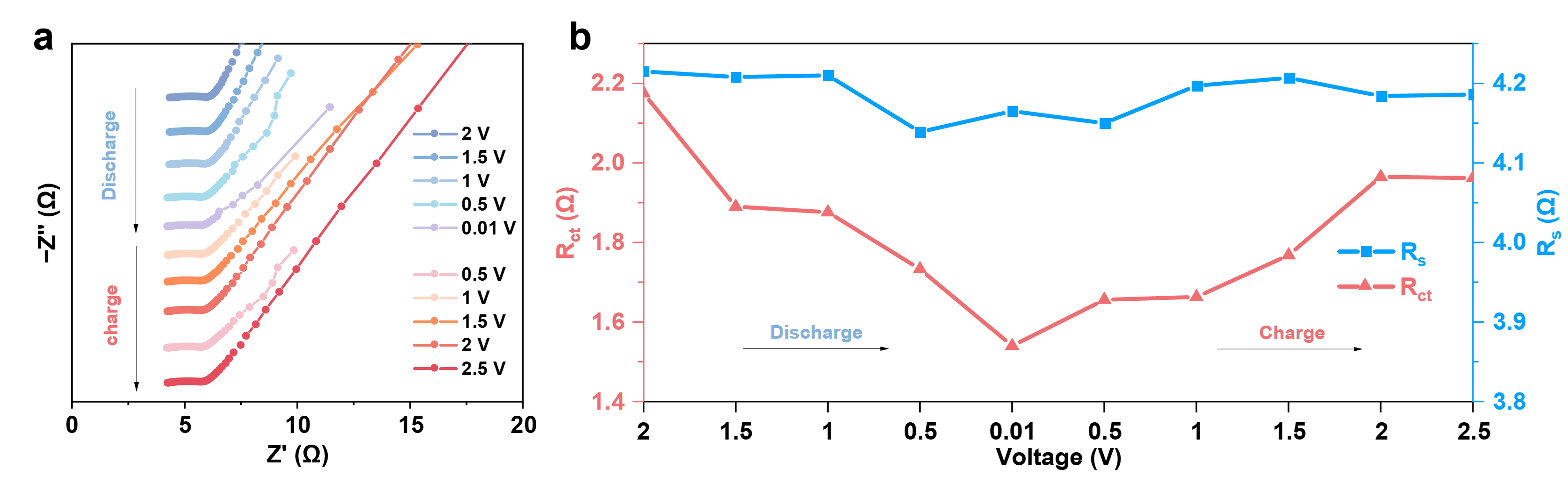


**Figure S17**. *In situ* electrochemical impedance spectroscopy (EIS) analysis of SAHTC-1300 during charge/discharge processes.


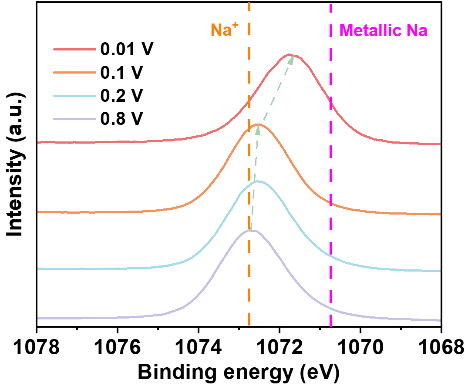


**Figure S18.** *Ex-situ* Na 1s XPS spectra for SAHTC-1300 electrode at different stage of sodiation during discharge.


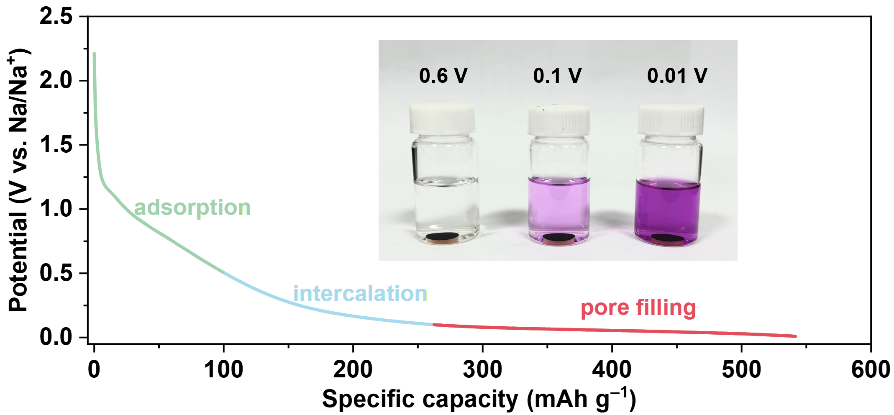


**Figure S19.** Optical photographs of electrodes discharged to various potentials after immersion in ethanolic phenolphthalein solution


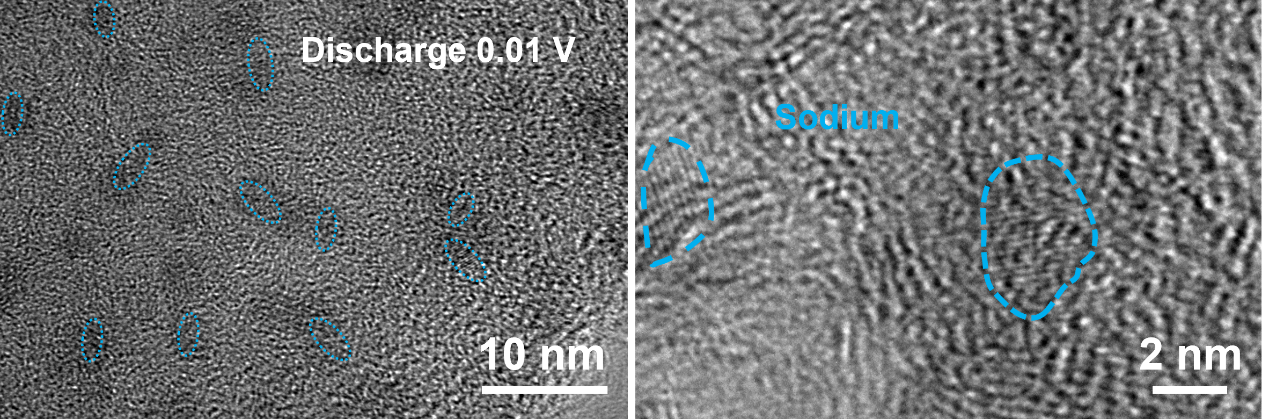


**Figure S20.** TEM images of the SAHTC-1300 electrode discharged to 0.01 V, along with corresponding close-up views.
